# Mesolimbic Dopamine Function Is Related to Salience Network Connectivity: An Integrative Positron Emission Tomography and Magnetic Resonance Study

**DOI:** 10.1016/j.biopsych.2018.09.010

**Published:** 2019-03-01

**Authors:** Robert A. McCutcheon, Matthew M. Nour, Tarik Dahoun, Sameer Jauhar, Fiona Pepper, Paul Expert, Mattia Veronese, Rick A. Adams, Federico Turkheimer, Mitul A. Mehta, Oliver D. Howes

**Affiliations:** a Department of Psychosis Studies, Institute of Psychiatry, Psychology and Neuroscience, Kings College London, De Crespigny Park, London, United Kingdom; b Department of Neuroimaging, Institute of Psychiatry, Psychology and Neuroscience, Kings College London, De Crespigny Park, London, United Kingdom; c Psychiatric Imaging Group, MRC London Institute of Medical Sciences, Hammersmith Hospital, London, United Kingdom; d Faculty of Medicine, Institute of Clinical Sciences, Imperial College London, London, United Kingdom; e Department of Mathematics, Imperial College London, London, United Kingdom; f EPSRC Centre for Mathematics of Precision Healthcare, Imperial College London, London, United Kingdom; g Institute of Cognitive Neuroscience, University College London, London, United Kingdom; h Division of Psychiatry, University College London, London, United Kingdom; i Department of Psychiatry, University of Oxford, Warneford Hospital, Oxford, United Kingdom

**Keywords:** ^18^F-DOPA, Functional connectivity, Graph theory, ^11^C-PHNO, Resting state, Striatum

## Abstract

**Background:**

A wide range of neuropsychiatric disorders, from schizophrenia to drug addiction, involve abnormalities in both the mesolimbic dopamine system and the cortical salience network. Both systems play a key role in the detection of behaviorally relevant environmental stimuli. Although anatomical overlap exists, the functional relationship between these systems remains unknown. Preclinical research has suggested that the firing of mesolimbic dopamine neurons may activate nodes of the salience network, but in vivo human research is required given the species-specific nature of this network.

**Methods:**

We employed positron emission tomography to measure both dopamine release capacity (using the D_2/3_ receptor ligand ^11^C-PHNO, *n* = 23) and dopamine synthesis capacity (using ^18^F-DOPA, *n* = 21) within the ventral striatum. Resting-state functional magnetic resonance imaging was also undertaken in the same individuals to investigate salience network functional connectivity. A graph theoretical approach was used to characterize the relationship between dopamine measures and network connectivity.

**Results:**

Dopamine synthesis capacity was associated with greater salience network connectivity, and this relationship was particularly apparent for brain regions that act as information-processing hubs. In contrast, dopamine release capacity was associated with weaker salience network connectivity. There was no relationship between dopamine measures and visual and sensorimotor networks, indicating specificity of the findings.

**Conclusions:**

Our findings demonstrate a close relationship between the salience network and mesolimbic dopamine system, and they are relevant to neuropsychiatric illnesses in which aberrant functioning of both systems has been observed.

SEE COMMENTARY ON PAGE 366

Resting-state functional magnetic resonance imaging (rfMRI) has demonstrated that activity within networks of brain regions is temporally correlated even in the absence of explicit external demands (1) and furthermore that these networks underlie human cognition and behavior 2, 3. The salience network, also referred to as the cingulo-opercular network, is centered around the anterior insula and dorsal anterior cingulate and in some instances has also been proposed to contain subcortical structures including the limbic (ventral) striatum and substantia nigra 4, 5. Recent meta-analyses synthesizing structural and functional imaging data have identified this network as uniquely affected across psychiatric disorders 4, 5.

The salience network plays a key role in identifying the most relevant internal and external stimuli to guide behavior appropriately 6, 7, 8, 9, 10, 11. Connectivity within the salience network is increased by externally directed demands, which contrasts with the default mode network (centered around the ventromedial prefrontal cortex and the posterior cingulate cortex) 12, 13, where connectivity is enhanced during self-generated thought 14, 15. The salience network dynamically coordinates the activity of other networks, in particular switching away from the default mode to task-positive networks when appropriate, and impaired communication between the default mode and salience networks is seen in a range of disorders, including schizophrenia, drug addiction, and cognitive impairment 16, 17, 18, 19, 20, 21.

Dopamine neurons also play a role in the identification of behaviorally relevant environmental stimuli. Mesolimbic dopamine neurons (projecting from the ventral tegmental area to the limbic striatum) have been proposed to signal reward prediction errors, which signal the discrepancy in the observed and predicted value of a stimulus (22). More recent research, however, has shown that these neurons respond to surprising stimuli even in the absence of any change in value, suggesting that their role extends to assigning salience to relevant environmental stimuli in general, not solely on the basis of value 23, 24. Dysfunction of this system is also observed in many neuropsychiatric illnesses 25, 26.

The need to develop an integrative understanding regarding the roles of the salience network and the mesolimbic dopamine system has been previously stressed (9). Given their overlap in function, it may be hypothesized that mesolimbic dopamine signaling plays a role in the modulation of the salience network. Recently, chemogenetic, optogenetic, and electrical stimulation of mesolimbic dopamine neurons in rodent models have been shown to activate salience network nodes, including regions not directly innervated by the ventral tegmental area 27, 28, 29, 30. While cross-species similarities exist in the organization of cortical networks, there are also marked differences. Longer distance connections in particular are proportionally much weaker in primates, potentially contributing to an increased vulnerability to “disconnection syndromes” such as schizophrenia (31). As a result, in vivo human research is required for a comprehensive understanding of the relationship between network connectivity and neurochemical signaling. Human studies have demonstrated effects of pharmacological dopaminergic challenges on salience network connectivity, suggesting that dopamine might regulate the salience network in humans, but, crucially, these studies are limited in their explanatory potential because of the nonphysiological and anatomically nonspecific effects of the intervention 32, 33, 34. Thus, it remains unclear whether mesolimbic dopaminergic signaling is linked to the salience network in humans.

To address this, we employed positron emission tomography (PET) to measure both dopamine synthesis capacity and dopamine release capacity, and rfMRI to evaluate salience and default mode networks at rest in the same participants. Based on recent preclinical findings that stimulation of dopamine neurons projecting to the limbic striatum activates regions of the salience network 27, 28, 29, our primary hypothesis was that individuals with greater striatal dopamine synthesis and release capacity would show greater connectivity within the salience network, and, because of the reciprocal relationship between salience and default mode networks, weaker connectivity within the default mode network (27).

In addition, we identified within these networks regions that played the most important role in information processing (“hub nodes”). Hubs support the rapid integration of information across a complex system and as such can be considered an optimal target via which a network input may efficiently maximize its influence in a coordinated fashion 35, 36. We therefore hypothesized that there would not be a uniform association between dopamine function and connectivity but that hub nodes would show the strongest association with dopamine function.

Given the preclinical emphasis on the mesolimbic dopamine projection, we focused on dopamine measures within the limbic striatum. However, we also explored the relationship between network connectivity and dopamine function in the associative and sensorimotor (dorsal) striatum. In addition, to provide a control condition, we investigated the relationship between striatal dopamine function and the visual and sensorimotor networks (networks not directly involved in salience processing), where we did not expect a relationship to be present.

## Methods and Materials

**Figure 1:**
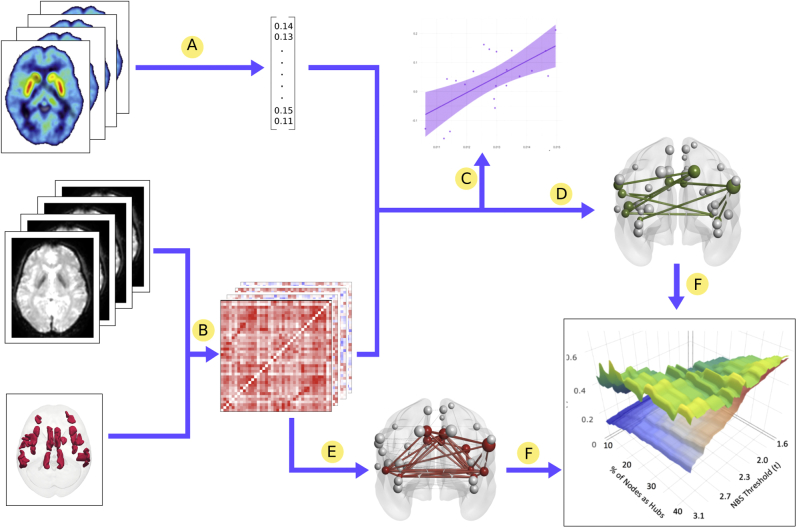
Summary of methods. **(A)***K*_i_^cer^ or change in nondisplaceable binding potential (ΔBP_ND_) obtained for each participant from positron emission tomography data. **(B)** Resting-state functional magnetic resonance imaging time courses extracted from salience network nodes and individual functional connectivity graphs constructed for each participant. **(C)** Relationship between salience network average strength and dopamine measure investigated. **(D)** Dopamine-associated subnetworks identified using network-based statistics (NBS). **(E)** Hub nodes identified from resting-state functional magnetic resonance imaging data. **(F)** Overlap between hub nodes and dopamine-associated subnetworks investigated.

**Figure 2:**
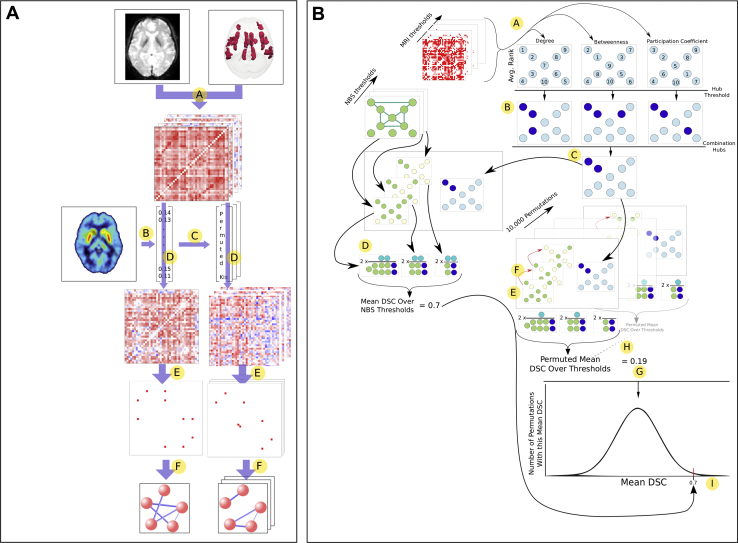
Methods for **(A)** identifying dopamine-associated nodes using the network-based statistic (NBS) and **(B)** identifying overlap between dopamine-associated nodes and hub nodes. Methods are described for ^18^F-DOPA and salience network but are identical for ^11^C-PHNO and other networks. A, Individual functional connectivity graphs constructed for the salience network from resting-state functional magnetic resonance imaging (rfMRI) data for each participant. B, Limbic *K*_i_^cer^ obtained for each participant from positron emission tomography (PET) data. C, *K*_i_^cer^ randomly permuted 10,000 times. D, Group PET-MRI graph constructed; each edge represents the correlation between that edge’s functional connectivity and the limbic *K*_i_^cer^ values (Ki). E, PET-MRI graphs thresholded and binarized. F, Number of edges of the largest connected component in both the actual PET-MRI graph (five edges in the illustrated example) and the permuted graphs (three edges in the example) compared. The *p* values were calculated based on the proportion of permuted examples the true example is larger than. A, Each node is ranked according to degree, betweenness centrality, and participation coefficient at each MRI threshold. The average rank across thresholds for each metric is then calculated. B, Nodes thresholded at a given rank; here the top-ranked 30% are chosen. C, Determine which nodes pass the threshold for all three metrics, here 20% of nodes classified as “combination hubs.” D, Calculate the Dice similarity coefficient (DSC) between the “combination hubs” and the nodes that form part of the dopamine subnetwork previously identified (Figure 1A). Do this for each NBS threshold and calculate the average DSC across thresholds. E, Randomly pick a selection of nodes equal in number to the number of nodes in the subnetwork at the most lenient NBS threshold. F, Match the number of nodes in this random selection to that in the subnetwork at more stringent thresholds by randomly deleting a node (red arrow) when necessary to match.

The experimental approach is summarized in Figures 1 and 2. PET was used to investigate two different aspects of dopaminergic functioning. In experiment 1, we measured dopamine synthesis capacity, while in experiment 2, we measured dopamine release capacity. rfMRI was used to investigate salience and default mode network connectivity. The relationship between salience and/or default mode connectivity and dopamine function was then investigated using a graph theoretical approach in which brain regions are represented as nodes and functional connections between these regions are represented as edges linking these nodes.

We first investigated whether network connectivity was associated with measures of dopamine function and identified specific nodes that were associated with dopamine function. We then separately classified nodes as information-processing hubs solely based on their pattern of rfMRI connectivity, and we determined whether dopamine-associated nodes overlapped significantly with these hub nodes.

In addition, the visual and sensorimotor networks were examined as control networks, as they are not directly involved in salience processing and show a lack of activation in preclinical studies of mesolimbic dopamine effects 27, 28, 29. Further details are given below and in the Supplement.

### Experiment 1: Dopamine Synthesis Capacity

Participants underwent a PET scan with the ligand 3,4-dihydroxy-6-[^18^F]fluoro-L-phenylalanine (^18^F-DOPA). ^18^F-DOPA PET measures the rate constant *K*_i_^cer^ for ^18^F-DOPA uptake, transport into synaptic vesicles, and its conversion into ^18^F-dopamine, thus providing a measure of dopamine synthesis capacity (37).

A region-of-interest analysis was performed to determine the limbic striatum influx constant (*K*_i_^cer^ [1/min]) (38). We also determined influx constants for associative and sensorimotor striatum, with these regions defined using the approach outlined by Martinez *et al.* (38).

Participants also underwent an rfMRI scan on a 3T GE Signa magnetic resonance scanner (GE Healthcare, Chicago, IL).

### Experiment 2: Dopamine Release Capacity

Participants underwent two PET scans with the D_2/3_ receptor ligand [^11^C]-(+)-4-propyl-9-hydroxy-naphthoxazine (^11^C-(+)-PHNO). A placebo scan gives a measure of baseline D_2/3_ receptor availability (nondisplaceable binding potential [BP_ND_]), while a scan following dexamphetamine administration allows quantification of the change in BP_ND_ due to competition from increased synaptic dopamine concentrations. The percentage reduction in D_2/3_ receptor availability between placebo and dexamphetamine scans thus provides an index of dopamine release capacity. We calculate the percent change in BP_ND_ as follows:

$$\Delta BP_{ND}=100\times \frac{BP_{ND\left(\mathrm{baseline}\right)}-BP_{ND\left(\mathrm{dexamphetamine}\right)}}{BP_{ND\left(\mathrm{baseline}\right)}}\%$$

Either placebo or 0.5 mg/kg dexamphetamine was administered orally 3 hours before ^11^C-(+)-PHNO administration, so that scan acquisition coincided with the expected time of peak action (39). ΔBP_ND_ was measured in the same regions as in experiment 1.

Participants also underwent an rfMRI scan using a Siemens MAGNETOM Verio 3T scanner (Siemens Corp., Erlangen, Germany).

### Common Methods

#### Participants

Participants had no previous or current history of psychiatric illness (assessed by the Structured Clinical Interview for DSM-IV Axis I Disorders).

#### Magnetic Resonance Imaging Analysis

Time series were extracted from 333 predefined nodes of interests of the Gordon cortical atlas. The salience and default mode network nodes of the Gordon atlas are displayed in Supplemental Figure S1. For each participant, a graph representing a functional connectivity network was constructed, each edge representing the level of functional connectivity between a pair of nodes. To demonstrate the robustness of our findings, we also replicated all analyses using two alternative atlases—the Power (40) and CONN network (41) atlases. Furthermore, in addition to using the a priori defined network labels for each node (e.g., salience, default mode), we also ran a whole-brain community detection algorithm for each atlas (42) to generate definitions of the salience and default mode networks based on the connectivity patterns present in the current data sets, and we repeated our analyses using these data-driven node assignments.

#### Network Strength and Dopamine Function

For each participant and each network, average network strength was defined as the mean z-transformed Pearson’s correlation coefficient between all network nodes (i.e., mean edge strength) (43). We first calculated Pearson correlation coefficients between network average strength and the PET measures of dopamine function. We then tested whether the correlation between network strength and dopamine function was significantly different between default mode and salience networks using the method described by Meng *et al.* as implemented in the cocor (1.1-3) package for R 3.3.2 44, 45. We also investigated the correlation between dopamine measures and salience–default mode “balance” (salience network average strength minus default mode network average strength).

#### Identifying Dopamine-Associated Nodes

To identify whether specific nodes show a significant relationship with limbic dopamine synthesis capacity, we used the network-based statistic to investigate salience, default mode, sensorimotor, and visual networks separately (see Figure 2A and Supplemental Methods) (46). Within each network, we identified subnetworks showing a significant relationship with dopamine function; we term these “dopamine-associated subnetworks,” and the nodes within these networks “dopamine-associated nodes.” In addition to examining intranetwork connectivity, we used the same approach to examine salience and default mode internetwork connectivity. To ensure the robustness of the results, this approach was undertaken across a range of network-based statistic thresholds (100 thresholds, *t* = 1.3–3.1, equivalent to *p* = .2–.005 for *n* = 23), where weaker thresholds will capture subnetworks showing a widespread diffuse relationship with dopamine function, and more stringent thresholds identify smaller clusters showing the strongest relationship.

#### Identifying Network Hubs

Based on the patterns of resting-state connectivity within the salience and default mode networks, we then calculated several graph metrics to identify network hubs. We calculated node degree (47)*,* betweenness centrality (47)*,* and participation coefficient (48)*.* We termed a node that ranked highly on all three metrics a “combination hub” (Figure 2B steps A–C), highlighting its importance as an all-round information-processing node. By varying the stringency of criteria used to defined nodes as hubs, we defined sets of combination hubs comprising between 10% and 40% of the total number of nodes.

#### Identifying Overlap Between Dopamine-Associated Nodes and Network Hubs

We next asked whether dopamine-associated nodes were statistically more likely to be combination hubs. We quantified the overlap of dopamine-associated nodes and combination hubs using the Dice similarity coefficient (where *A* is the set of dopamine-associated nodes and *B* is the set of combination-hub nodes) 49, 50:

$$Dice\;Similarity\;Coefficient=\frac{2\left|A\cap B\right|}{\left|A\right|+\left|B\right|}$$

The Dice coefficient was calculated for each of the 100 network-based statistic thresholds (*t* = 1.3–3.1) and then averaged to give a single score (Figure 2B part D). Permutation testing was used to test whether this overlap score was statistically significant. This procedure was then repeated for each of the combination-hub thresholds (10%–40%), thereby giving a *p* value for each hub threshold.

We also investigated whether there was a significant overlap between ^18^F-DOPA and ^11^C-(+)-PHNO dopamine-associated nodes.

## Results

### Participants

Twenty-one participants took part in experiment 1, the ^18^F-DOPA study (mean [SD] age = 23.5 years [3.36 years]; 67% male). Twenty-three participants took part in experiment 2, the ^11^C-(+)-PHNO study (mean [SD] age = 24.4 years [4.5 years]; 57% male).

### Network Strength and Dopamine Function

#### Experiment 1: Dopamine Synthesis Capacity (^18^F-DOPA)

**Figure 3:**
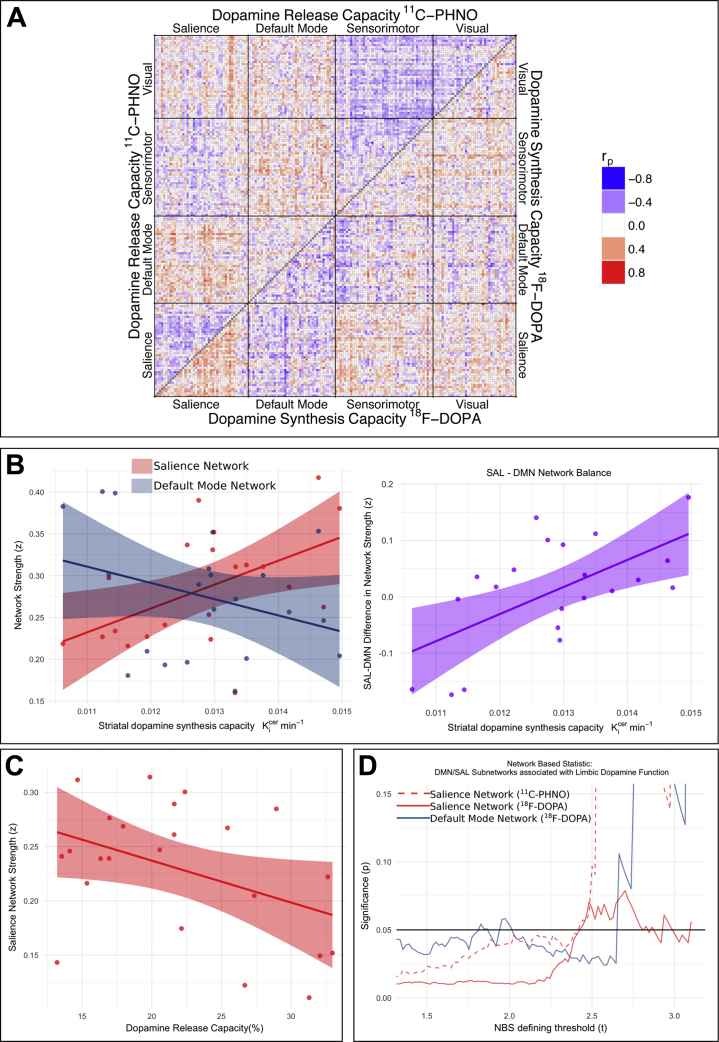
Resting-state networks and their relationship with limbic dopamine function. **(A)** Dopamine-associated graphs—each edge represents the correlation between that edge’s resting-state functional magnetic resonance imaging functional connectivity values and limbic dopamine synthesis and/or release capacity. **(B)** Dopamine synthesis capacity is correlated with salience network (SAL) strength (*n* = 21, *r*_p_ = .51, *p* = .017), did not correlate with default mode network (DMN) strength (*r*_p_ = −.32, *p* = .16), and is positively correlated with the difference between SAL strength and DMN strength (*r*_p_ = .60, *p* = .004). **(C)** Dopamine release capacity negatively correlated with SAL strength (*r*_p_ = −.42, *p* = .049). **(D)** Network-based statistic (NBS) identifies subnetworks significantly associated with dopamine synthesis and/or release capacity across a range of thresholds.

The correlations between edge strength and limbic dopamine synthesis capacity are displayed in the lower triangle of Figure 3A. Average network strength of the salience network positively correlated with limbic dopamine synthesis capacity (*r*_p_ = .51, *p* = .017, Figure 3B), and this was also significant for all other parcellations (*r*_p_ = .44–.62) (Supplemental Figure S3). In contrast, average network strength of the default mode network did not show a significant relationship with limbic dopamine synthesis capacity (*r*_p_ = −.32, *p* = .16) (Figure 3B).

The correlation between dopamine synthesis capacity and salience network average strength was significantly different from that between dopamine synthesis capacity and default mode average network strength (*z* = −2.7, *p* = .008). Furthermore, salience–default mode balance (salience network average strength minus default mode network average strength) correlated with dopamine synthesis capacity (*r*_p_ = .60, *p* = .004) (Figure 3B).

When the relationship between salience network average strength and dopamine synthesis capacity in other striatal regions was investigated, the findings were significant for the associative striatum (*r*_p_ = .46, *p* = .034) but not the sensorimotor striatum (*r*_p_ = .43, *p* = .053) (Supplemental Figure S2). As hypothesized, there was no association between limbic dopamine synthesis capacity and average network strength of either the visual (*r*_p_ = .05, *p* = .85) or sensorimotor (*r*_p_ = .09, *p* = .68) networks.

#### Experiment 2: Dopamine Release Capacity [^11^C-(+)-PHNO]

The correlations between edge strength and limbic dopamine release capacity are displayed in the upper triangle of Figure 3A. Contrary to our hypothesis, average network strength of the salience network was negatively correlated with limbic dopamine release capacity (*r*_p_ = −.42, *p* = .049) (Figure 3B), a finding that was also significant for some (Gordon data–driven, Power data–driven) but not all (Power a priori, CONN) of the alternative parcellations (*r*_p_ = −.24 to −.52) (Supplemental Figure S3). There was no significant correlation between dopamine release capacity and default mode average network strength (*r*_p_ = .03, *p* = .9). The difference between these two correlations was not significant (*z* = 1.43, *p* = .15), and salience–default mode balance did not correlate significantly with dopamine release capacity (*r*_p_ = −.29, *p* = .18).

As in experiment 1, salience network strength was significantly associated with dopamine release capacity in the associative striatum (*r*_p_ = −.5, *p* = .015) but showed no relationship with the sensorimotor striatum (*r*_p_ = −.17, *p* = .44) (Supplemental Figure S2). Furthermore, as in experiment 1, there were no associations between limbic dopamine release capacity and average network strength in either the visual (*r* = −.27, *p* = .22) or sensorimotor (*r* = −.28, *p* = .20) networks. Interestingly, however, in an exploratory analysis, dopamine release capacity within the sensorimotor striatum showed a significant relationship with sensorimotor network average strength (*r* = −.58, *p* = .004).

There was no relationship between rfMRI motion and either network strength or dopamine measures (results in the Supplement).

### Identifying Dopamine-Associated Nodes

#### Experiment 1: Dopamine Synthesis Capacity (^18^F-DOPA)

Using the network-based statistic, we identified salience network subnetworks showing a significant positive relationship with limbic dopamine synthesis capacity across a range of thresholds (Figure 3D). In contrast, subnetworks within the default mode network showed a significant negative relationship with dopamine synthesis capacity.

We also used the network-based statistic to examine internetwork connections between default mode and salience networks. At specific thresholds, greater dopamine synthesis capacity was associated with weaker internetwork connectivity (i.e., greater decoupling), although this was not significant across a wide range of thresholds (Supplemental Figure S4).

When dopamine synthesis capacity in other striatal subdivisions was examined, the findings were again significant for the associative but not sensorimotor striatum (Supplemental Figure S5). The specificity of the findings was again demonstrated by the fact that no dopamine-associated subnetworks were identified in either visual (*p* > .29 for all thresholds) or sensorimotor (*p* > .38) networks.

#### Experiment 2: Dopamine Release Capacity [^11^C-(+)-PHNO]

We identified subnetworks within the salience network showing a significant negative relationship with limbic dopamine release capacity (Figure 3D). No default mode subnetworks showed a significant association with dopamine release capacity. As in experiment 1, examination of internetwork connections suggested that release capacity was associated with internetwork coupling only at specific thresholds, and in this case greater release capacity was associated with stronger coupling (Supplemental Figure S4).

Dopamine release in other regions was examined, and similarly to experiment 1, significant results were observed for the salience network with the dopamine measure in the associative striatum but not sensorimotor striatum (Supplemental Figure S5). As before, we demonstrated the specificity of findings in that no visual (*p* > .12 all thresholds) or sensorimotor subnetwork (*p* > .11 all thresholds) was associated with limbic dopamine release capacity.

In both experiments, these findings were seen in various parcellations and methods of node assignment (Supplemental Figure S6).

### Identifying Overlap Between Dopamine-Associated Nodes and Network Hubs

#### Experiment 1: Dopamine Synthesis Capacity (^18^F-DOPA)

**Figure 4:**
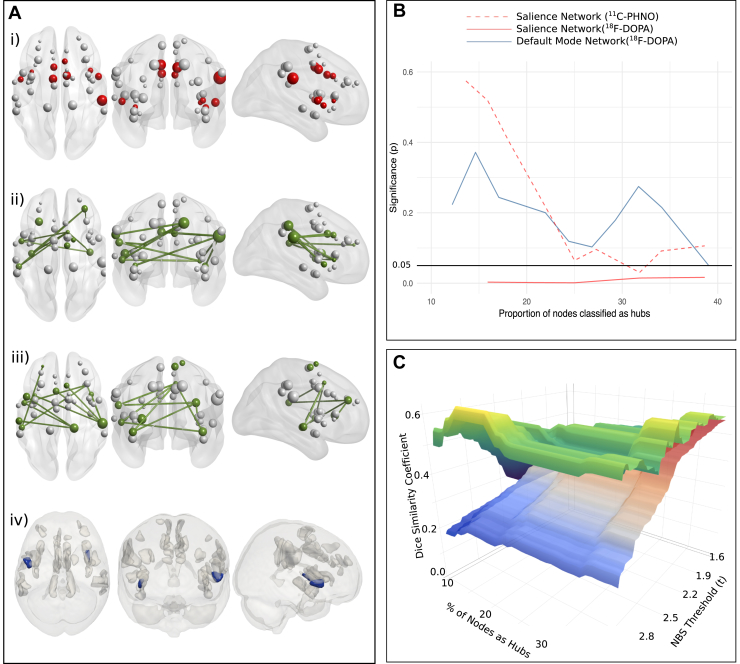
Characterization of dopamine-associated subnetworks. **(A)** Salience-network hubs and dopamine-associated subnetworks: red nodes represent network combination hubs and green nodes and edges represent the dopamine-associated network, in which edge strength correlates with limbic dopamine synthesis capacity. i) Hub nodes in experiment 1; ii) dopamine-associated network in experiment 1 at a specific network-based statistic (NBS) threshold; iii) dopamine-associated network in experiment 2; iv) Two nodes classified as both dopamine-associated nodes and hub nodes in both experiments at the most stringent threshold. **(B)** Graph displaying whether overlap between dopamine-associated nodes and combination hubs is significant. **(C)** Illustrating the overlap between salience network hub nodes and nodes involved in for ^18^F-DOPA-associated subnetworks. The top (green-yellow) layer represents the Dice similarity coefficients for the observed dopamine-associated nodes and the combination-hub nodes, while the bottom (blue-red) layer represents the mean overlap coefficients of 10,000 randomized networks. In this figure, the Dice coefficient is plotted individually for each NBS threshold (i.e., not averaged as in the construction of Figure 3B).

We next investigated whether the dopamine-associated nodes identified in the previous step overlapped significantly with nodes that were classified as information-processing hubs. Within the salience network, we found that regardless of how many nodes were defined as hubs within our range of investigation (i.e., the top-ranked 10%–40%), these nodes were likely to be dopamine-associated nodes, and this overlap was significantly more likely than expected by chance for all hub thresholds (Figure 4B), and this was the case for all parcellations and methods of node assignment (Supplemental Figure S7). The Dice coefficient between nodes in salience–^18^F-DOPA subnetworks and combination hubs across a range of thresholds is shown in Figure 4C, illustrating that the nodes that are most strongly associated with dopamine synthesis capacity (i.e., those surviving the more stringent network-based statistic thresholds) are also the most likely to be key information-processing hubs (as defined by resting-state functional connectivity).

The Dice coefficient between combination hubs and the dopamine-associated nodes within the default mode network was numerically greater than the Dice coefficient of the random network at all thresholds, but this difference was statistically significant only for certain hub thresholds and parcellations (see Figure 4B and Supplemental Figure S7).

#### Experiment 2: Dopamine Release Capacity [^11^C-(+)-PHNO]

The Dice coefficient between combination hubs and the salience–^11^C-(+)-PHNO subnetworks were numerically greater than the mean overlap expected of the random network, but this difference was statistically significant only for certain thresholds and parcellations (Figure 4B and Supplemental Figure S7).

#### Overlap Between Experiments

Dice overlap scores between the ^11^C-(+)-PHNO– and ^18^F-DOPA–associated nodes ranged from 0.36 at the most stringent network-based statistic threshold where equal node networks existed (number of nodes = 11) to 0.92 at the least stringent threshold (number of nodes = 36). None of these overlaps was greater than would be expected by chance (*p* > .20 for all thresholds). We then investigated which nodes were in dopamine-associated networks at the most stringent threshold and were also combination hubs (ranked in the top 11/40 nodes in both experiments). Only two nodes fulfilled these criteria; these were located bilaterally in the insula (see Figure 4A part iv).

## Discussion

Using rfMRI and a dual-tracer PET paradigm, we demonstrate a strong relationship between limbic dopamine function and salience network functional connectivity in humans. Both the salience network and mesolimbic dopamine system are central to the pathophysiology of various neuropsychiatric disorders 4, 5, 25, 26. To our knowledge, however, this is the first human study to both measure limbic dopamine function and investigate its relationship with the salience network.

Specifically, we demonstrated that stronger connectivity within the salience network was directly associated with limbic dopamine synthesis capacity and, contrary to our initial hypothesis, was inversely associated with limbic dopamine release capacity. Furthermore, the biological relevance of this result is supported by the finding that there was significant overlap between nodes in salience subnetworks associated with dopamine synthesis capacity and nodes separately identified as information-processing hubs. We also identified default mode subnetworks in which edge strength was inversely correlated with synthesis capacity.

### The Relationship Between Mesolimbic Dopamine Function and the Salience Network

The current study advances our understanding regarding the relationship between mesolimbic dopamine activity and salience network function. Preclinical studies have suggested a link between mesolimbic dopamine function and nodes of the salience network 27, 28, 29. However, a precise homologue of the salience network is not present in rodent models, because of both the species-specific nature of cortical networks and the fact that in humans the network is characterized by the presence of Von Economo neurons, a distinct set of pyramidal neurons, which are not observed in rodents 31, 51, 52. Previous studies in humans have used rfMRI in combination with pharmacological manipulations of the dopamine system 32, 53, 54, 55. Without the use of PET, however, it is not possible to obtain a measure of the dopaminergic effect of the pharmacological intervention, which can vary significantly between individuals for the same dose. Furthermore, drug challenges perturb the system widely, causing various neurochemical changes across the brain and affecting neurovascular coupling (56). In contrast, our resting-state data were obtained in a drug-free state, and ^18^F-DOPA PET indexes physiological dopamine function.

Although previous studies have integrated PET and the examination of resting-state networks, these have predominantly obtained only measures of baseline dopamine receptor availability 57, 58, 59, 60. Two studies have measured dopamine function but did not examine the relationship with the salience network 34, 61.

### Dopamine-Synthesis and Release Capacity

We hypothesized that release and synthesis capacity would capture similar facets of a single construct—the activity of an individual’s mesolimbic dopamine system. Our finding of divergent relationships between these two measures and salience network connectivity does not support this interpretation. Both release and synthesis capacity are complex signals, and the relationship between the two is not clear 62, 63. Synthesis capacity represents the rate of 3,4-dihydroxy-L-phenylalanine decarboxylation, and it depends on the number of dopaminergic neurons and their mean firing rate. Measures of release capacity will be determined by the reactivity of mesolimbic dopamine neurons to the effects of amphetamine. Agonist tracers such as ^11^C-(+)-PHNO preferentially bind to the high-affinity state of the D_2_ receptor 64, 65. This means that our measure of percentage release could be affected by the proportion of D_2_ receptors in a high-affinity state as well as the level of dopamine release. Future studies combining antagonist and agonist radiotracers would help determine the potential influence of interindividual differences in the proportion of D_2_ receptors in the high-affinity state. A tentative hypothesis that unites our findings assumes that in a healthy individual, dopamine synthesis capacity reflects a summary measure of tonic dopamine neuron firing and appropriate adaptive phasic firing, while release capacity reflects that individual’s propensity for spontaneous phasic firing in the absence of behaviorally relevant stimuli (66). Taken with the finding that reduced salience network connectivity is observed in disorders of aberrant salience processing, this suggests a model in which greater salience network connectivity is associated with the appropriate attribution of salience, mediated by robust adaptive dopaminergic signaling, while a propensity for stimulus-independent dopamine neuron firing is associated with weakening of the network and misattribution of salience 67, 68, 69, 70, 71. This is a speculative interpretation, however, and assumes that the consequences of higher dopamine synthesis capacity in healthy participants differ from those in patient populations where it has been linked to disorders of salience (72).

### Dopamine Pathways

Preclinical research has often focused on the dopamine neurons of the ventral tegmental area. The limbic striatum is a major projection target for these neurons and, as such, an appropriate region of focus. In rodents, however, the mesolimbic pathway is proportionally larger than in humans, and therefore, although the associative striatum receives dopaminergic innervation from the nigra, parts of the human midbrain-associative striatum pathway are homologous to the rodent mesolimbic pathway 73, 74. As a result, it is not surprising that the relationship observed between the salience network and limbic dopamine function was also seen when using measures of associative striatum dopamine function. No relationship, however, was seen with dopamine measures obtained from the sensorimotor striatum and salience network connectivity, although an association was seen with release capacity in this region and sensorimotor network connectivity, suggesting a degree of functional specificity in the relationship between dopamine measures and network connectivity.

### Clinical Implications

Structural and functional abnormalities within the salience network are a common biological substrate of mental illness and exist transdiagnostically across a broad range of disorders, including depression, schizophrenia, and Parkinson’s disease 4, 5, 75, 76, 77. The mesolimbic dopamine system is also affected in these disorders 25, 26, 78, 79. Our finding that the two systems show coupling in humans could explain why they are disordered in several illnesses, as dysfunction in any one of the network’s nodes could conceivably lead to impairment across both systems. Our findings also highlight opportunities for the development of pharmacological interventions. The ability to link the effects of quantifiable neurochemical modulation to change in network function raises the possibility of mapping receptor actions to desired network alterations.

### Limitations and Further Questions

Given that the firing of mesolimbic neurons has been shown to provoke widespread neural activity in regions receiving no direct dopaminergic innervation (29), our findings could be interpreted as indicating that mesolimbic dopaminergic signaling is able to regulate salience network function. However, while differences in dopaminergic tone could feasibly shift the balance between the two networks, it is not possible for us to infer the direction of causality.

Likewise, the precise site of relevant dopaminergic activity is not clear. ^11^C-(+)-PHNO and ^18^F-DOPA are unable reliably characterize dopamine function outside of the striatum, and it was therefore not possible to test whether a relationship between direct dopaminergic innervation of network nodes and network connectivity also existed.

We used an eyes-closed resting-state scan, and some networks have shown greater reliability when participants have kept eyes open. The differences are relatively small, however, and therefore unlikely to have significantly influenced our findings (80).

The use of acute dopaminergic challenges during simultaneous PET and MRI would allow for the study of intraindividual effects of dopaminergic release on network average strength and organization and may help to disentangle some of these issues. Studies in clinical populations, such as individuals with schizophrenia, where measures of both dopaminergic and network function may show wider ranges (81), and the inclusion of behavioral tests would help further determine the relevance of these findings to pathophysiology and psychopathology.

Measures of dopamine function showed strong associations with salience network connectivity, and in the case of dopamine synthesis capacity, this was particularly the case for nodes that were identified as information-processing hubs within the salience network. These findings are relevant to developing integrated models of brain function in health and disease and for the development of treatments that attempt to restore network function via neurochemical modulation.
