# Supplementary material for: Mesolimbic Dopamine Function Is Related to Salience Network Connectivity: An Integrative Positron Emission Tomography and Magnetic Resonance Study
Source: Biol Psychiatry. 2019 Mar 1;85(5):368–78. doi: 10.1016/j.biopsych.2018.09.010 (PMC6360933; doi:10.1016/j.biopsych.2018.09.010)
Supplement: Supplemental Material [file mmc1.pdf]

Mesolimbic Dopamine Function Is Related to Salience Network Connectivity: An Integrative Positron Emission Tomography and Magnetic Resonance Study

Supplementary Information

Table of Contents

Supplemental Methods **2**
- **Participants** **2**
- **PET Data Acquisition and Analysis** **2**
  - *Experiment 1: FDOPA Study* 2
  - *Experiment 2: PHNO Study* 3
- **MRI Data Acquisition** **3**
  - *Experiment 1: FDOPA Study* 3
  - *Experiment 2: PHNO Study* 4
- **fMRI Preprocessing** **4**
- **Graph Analysis** **5**
  - *MRI-analysis: Atlas Selection* 5
  - *MRI-analysis: Network Strength* 5
  - *MRI-analysis: Community Detection* 5
  - *Identifying Dopamine Associated Nodes– Network Based Statistic* 6
  - *Hub Node Identification* 7
  - *Identifying Overlap Between Dopamine Associated Nodes and Network Hubs* 8
- **Software** **9**

Supplemental Results **10**

Figure S1: Salience and default mode networks of the Gordon atlas **11**

Figure S2: Correlation between average network strength (Gordon parcellation) and dopamine measures in striatal subdivisions **12**

Figure S3: Correlation between limbic dopamine measures and salience network strength for a range of parcellation schemes **13**

Figure S4. Relationship between salience-default internetwork connectivity and limbic dopamine function **14**

Figure S5: Network-based statistic results for and dopamine measures in various regions (Gordon parcellation) **15**

Figure S6: Network based statistic identifies subnetworks significantly associated with limbic dopamine measures for a range of parcellations **16**

Figure S7: Significance of overlap between nodes showing strongest association with limbic dopamine measures and network combination hubs, for a range of parcellations . **17**

Supplemental References **18**

Supplemental Methods

Participants

Ethical permission was obtained from the local ethics committee, and all participants provided informed written consent. Healthy controls were recruited via advertisements online and in local media. Subjects had no history of psychiatric or neurological disorders, and had a urine drug screen and pregnancy test (where appropriate) prior to scanning.

PET Data Acquisition and Analysis

Participants were not permitted to smoke or consume caffeine for four hours preceding the scan. After acquiring a CT scan for attenuation correction, PET images were acquired using a Siemens Biograph HiRez XVI PET scanner (Siemens Healthcare, Erlangen, Germany) at Imanova Centre for Imaging Sciences.

Experiment 1: FDOPA Study

One hour prior to scanning, participants received 400mg entacapone and 150mg carbidopa, to prevent formation of radiolabelled metabolites and reduce peripheral metabolism. Approximately 160 MBq of ^{18}F-DOPA was administered by bolus intravenous injection. The quantification pipeline was consistent with previous works.[1] Correction for head movement during the scan was performed by denoising the non- attenuation-corrected dynamic images using a level 2, order 64 Battle-Lemarie wavelet filter. Frames were realigned to a single reference frame, acquired 20 minutes post-injection, employing a mutual information algorithm.[2,3] The transformation parameters were then applied to the corresponding attenuated-corrected dynamic images, creating a movement-corrected dynamic image, which was used in the analysis. Realigned frames were then summated to create an individual motion-corrected reference map for the brain tissue segmentation. The cerebellum was used as a reference region, and K_i^{cer} was calculated with the Patlak-Gjedde graphical approach adapted for reference tissue input function[4]. Image processing and quantification was done using in-house code with MATLAB 2012b.

Experiment 2: PHNO Study

Approximately 170 MBq of ^{11}C-(+)-PHNO was administered by bolus injection. After the administration of the radiotracer, dynamic emission data were acquired continuously for 90 minutes. The dynamic images were reconstructed using a filtered back-projection algorithm into 31 frames (8 x 15 seconds, 3 x 60 seconds, 5 x 120 seconds, 15 x 300 seconds) with a 128 matrix, a zoom of 2.6 and a transaxial Gaussian filter of 5mm.

An individual parcellation of the brain was implemented in MIAKAT release 4.2.6 (http://www.miakat.org),[5] SPM12 and FSL (version 5.0.9). Cerebellar grey matter was used as the reference region, and the simplified reference tissue model (SRTM) was used to derive BP_{ND} from the regional time activity curves.[6,7] The magnitude of dexamphetamine-induced dopamine release within the limbic striatum was quantified as the percentage change in BP_{ND} in the dexamphetamine condition vs. baseline (no dexamphetamine) condition.

$$\Delta BP_{ND} = 100 \cdot \frac{BP_{ND\,(baseline)} - BP_{ND\,(dexamphetamine)}}{BP_{ND\,(baseline)}} \%$$

MRI Data Acquisition

Participants were instructed to remain still, keep awake, and keep their eyes closed.

Experiment 1: FDOPA Study

MRI data was obtained using a General Electric (Milwaukee, Wisconsin, USA) Signa HDxt 3T magnetic resonance imaging system. Functional imaging consisted of T2* weighted echo planar image slices. 256 volumes were acquired, consisting of 39 interleaved slices (3.5 mm slice thickness, 3.75 mm x 3.75 mm voxel dimensions in plane) with a repetition time (TR) of 2000 ms, echo time (TE) of 30 ms, and a scan time of 8 minutes 32 seconds.

A structural image was obtained using a gradient-echo scan (TR=7.0s, TE=2.8s, flip angle=11°, in plane resolution=1mm x 1mm, slice thickness=1.2mm, 196 slices).

Experiment 2: PHNO Study

MRI data was obtained using a Siemens MAGNETOM Verio 3-T magnetic resonance imaging scanner. Functional imaging involved a multiband sequence based on the multiband EPI WIP v012b provided by the University of Minnesota,[8–11] using a multiband acceleration factor of 2. 238 volumes were acquired, consisting of 72 interleaved slices (2mm thickness, and in-plane resolution of 3 x 3 mm), with a TR of 2000 ms, TE of 30 ms and a scan time of 7 minutes 56 seconds.

A structural image was also obtained using a gradient echo scan (TR = 2300 ms, TE = 2.98 ms, flip angle = 9°, 1 mm isotropic voxels, parallel imaging (PI) factor =2, 160 slices).[12]

fMRI Preprocessing

Image pre-processing was performed via the CONN toolbox (version 17.b)[13] for Statistical Parametric Mapping software (SPM 12 (6906)). A standard preprocessing pipeline was used consisting of slice timing correction, realignment, and normalisation to MNI space. Images were smoothed with a Gaussian kernel of 8mm full-width-half-maximum. The ART toolbox was used to account for motion and artefact detection using anatomical component based correction (aCompCor) of temporal confounds relating to head movement and physiological noise. This method models noise effects at a voxel level based on estimates derived from principal components of noise regions of interest (white matter and CSF, eroded by one voxel to minimise partial volume effects), and then removes these from the BOLD timeseries using linear regression. Six residual head motion parameters and their first order temporal derivatives were also entered as regressors into the first level model. A confounding effect accounting for magnetisation stabilisation, and its first order derivative was entered. Artifact/outlier scans (average intensity deviated more than 5 standard deviations from the mean intensity in the session, or composite head movement exceeded 0.9 mm from the previous image) were also regressed out. Preprocessed data were temporally bandpass filtered (0.008-0.09 Hz)

Time-series were extracted from *N*=333 predefined nodes of interests of the Gordon cortical atlas. The salience and default mode network nodes of the Gordon atlas are displayed in Figure S1. For each participant, a graph representing a functional connectivity network was

constructed, each edge representing the level of functional connectivity between a pair of nodes, which was computed as the z-transformed Pearson's correlation coefficient between their mean time-series.

Graph Analysis

MRI-analysis: Atlas Selection

The Gordon parcellation is based upon resting state boundary maps observed in a sample of 120 healthy young adults, and shows superior within parcel homogeneity when compared to other parcellations, making it an ideal choice for the analysis of resting state data.[14] In order to demonstrate robustness of our findings, we also undertook all analyses using two alternative atlases - the Power atlas (a collection of 264 10 mm diameter spheres derived from connectivity data in over 300 healthy volunteers performing various tasks),[15] and the CONN network atlas (a 32 node atlas in which nodes are defined on the basis of an independent components analysis of 497 subjects from the Human Connectome Project).[13]

MRI-analysis: Network Strength

For a network formed of N nodes, the average network strength $\bar{s}$ can be computed similarly to the link density ρ of unweighted networks[16]: $\bar{s} = 2/N(N-1)\sum_{i,j=1}^{N} w_{ij}$.

MRI-analysis: Community Detection

On the basis of the original Gordon atlas labels 41 nodes were a priori defined as belonging to the default mode network, and 44 to the cinguloopercular/salience network (referred to in the current paper as the salience network).[14] The Power atlas assigns 32 and 58, while the CONN atlas assigns 7 and 4 nodes to the salience and default mode networks respectively. As a result, the networks of interest were defined across a wide range of scales both in terms of node volume, and maximal network size.

In addition to the apriori network labels, however, we also ran a whole brain community detection algorithm for each atlas,[17] to generate definitions of the salience and default mode networks based on the connectivity patterns present in the current datasets. To accomplish this each subject's fully weighted functional connectome was subjected to the Louvain

community detection algorithm, and the results of this were used to generate community assignments at the group level (individual level community assignments are not appropriate for subsequent analyses).[17] Due to the non-deterministic nature of the Louvain algorithm, a previously described consensus clustering approach was employed,[18] negative weights were treated symmetrically, and the gamma parameter was set to 1.7 as this produced community sizes in relative agreement with existing parcellation schemes.

Identifying Dopamine Associated Nodes– Network Based Statistic

In order to identify whether specific subnetworks show a significant relationship with limbic dopamine synthesis capacity we used the Network-Based Statistic (NBS) to investigate salience, default mode, sensorimotor, and visual networks separately (the method is summarised in Figure 2A in the main text).[19] A t-statistic was generated for each edge based on the Pearson correlation coefficient computed over each population between the functional connectivity z values of that edge, and the limbic striatum K_i^{cer} or ΔBP_{ND} values (i.e. a positive value indicates that greater limbic dopamine synthesis/release capacity is associated with stronger connectivity at that edge). This generated a group level PET-MRI graph for each cohort, which was thresholded at 100 separate thresholds (t=1.3-3.1, equivalent to p=0.2-0.005 for n=23). The connected component with the greatest density (i.e. greatest number of edges) within this t-statistic graph was then determined at each of these t-thresholds. If there were multiple networks of equal density the one with greatest average strength in the weighted version of the graph was selected. Permutation testing was performed to calculate a p-value for each threshold, by determining whether the density of the densest component was significantly greater than expected by chance. This was calculated by comparing the density of the observed component with densest components in 10,000 PET-MRI graphs generated for each threshold (t=1.3-3.1) via random assignment of the K_i^{cer} or ΔBP_{ND} values. We subsequently term the components identified as showing a relationship with limbic dopamine function as 'dopamine-associated subnetworks'. We repeated the analysis using measures for dopamine function in the associative and sensorimotor striatum in place of the limbic measure. We used the same approach to examine inter-network connectivity between salience and default mode networks.

Hub Node Identification

Group level binary graphs were constructed for the salience and default mode networks separately. First individual level graphs were rescaled by subtracting from each individual participant's graph that participant's average network strength, and dividing by the standard deviation of all that network's edges' strength. In this way all participants then had an average network strength of zero and so individuals with greater network mean strength would not have undue topological influence.

We next averaged across individuals to create a group level graph. Proportional thresholding was performed on this group averaged matrix by assigning a value of 1 to all edges with connection strength above a set threshold, and setting all remaining edges to 0. We used 100 thresholds, retaining 20% of edges at the most lenient threshold, and 7% at the most stringent. There is no 'correct' set of thresholds but at more lenient thresholds one risks including a high degree of spurious connections, while at more stringent thresholds the graph became overly fragmented. The fact that this is a more lenient range than reported elsewhere is appropriate given we are investigating intranetwork connectivity, where there will be a lower proportion of spurious edges.[20]

Graph metrics were computed using the Brain Connectivity Toolbox.[21] Node *degree* k_i refers to the number of neighbours a node has, and is thus a measure of the local, direct importance of a node: $k_i = \sum_{j=1}^{N} A_{i,j}$.[22] While this intuitively captures the relative importance of a node within a network, in correlation based graphs, it may also reflect membership of a larger community, as opposed to the importance of the node in information processing.[23] We therefore also calculated for each node *betweenness centrality* B_i,[22] and the node - *participation coefficient* PC_i.[24] The node betweenness centrality measures the proportion of shortest paths between all pairs of nodes that pass through it, and reflects its position as a potential information broker in the network. It is formally defined as: $B_i = \sum_{m,n=1|i \neq m \neq n}^{N} \frac{|\sigma_{m,n}(i)|}{|\sigma_{m,n}|}$, with $|\sigma_{m,n}|$ the number of shortest paths between nodes *m* and *n*.[22] The node participation coefficient was calculated after first assigning each node to a community using the Louvain community detection algorithm.[17] A participation coefficient of zero means that all the edges of a node are restricted to its own community, indicating a

rather local role, whereas a value approaching 1 means that its edges are evenly distributed among all the communities of the graph – indicating that the node plays a role in integrating different clusters of the graph. The participation coefficient is defined as $PC_i = 1 - \sum_{C=1}^{N_c} \left(\frac{k_i^C}{k_i}\right)^2$, with N_C the number of communities and k_i^C, the degree of a node restricted to community C.

At each MRI threshold, every node was ranked on each of these metrics, and the mean rank of each node across MRI thresholds was then calculated. We then set a rank threshold, and for each metric selected only the nodes ranking above it. If any node ranked above this threshold for all three metrics it was termed a *combination hub* (main text Figure 2B steps A-C), highlighting its importance as an all-round information processing node. By next lowering the rank threshold we gradually increased the number of nodes meeting combination hub criteria, and so defined sets of combination hubs comprising between 10 and 40% of the total number of nodes. In some cases it is possible a specific hub threshold might have no eligible nodes (e.g. in the main paper figure 4B – the ^{18}F-DOPA salience network does not have combination until the threshold reaches 15%).

Identifying Overlap Between Dopamine Associated Nodes and Network Hubs

After identifying nodes within the default mode and salience networks that showed an association with measures of limbic dopamine function, we sought to identify whether these dopamine associated nodes tended to overlap with nodes classified as combination hubs (as defined above using the rfMRI data).

The overlap of dopamine associated nodes and combination hubs was quantified using the Dice Similarity Coefficient:[25,26]

$$Dice\ Similarity\ Coefficient = \frac{2|A \cap B|}{|A| + |B|}$$

A is the set of nodes in the dopamine associated subnetwork and B is the set of combination hub nodes. The Dice Coefficient was calculated for each of the 100 NBS thresholds (t=1.3-3.1) and then averaged to give a single 'true' score (main text Figure 2B part D). We then randomly

selected an assortment of nodes, equal in number to the number of nodes present in the most leniently thresholded original network-based statistic subnetwork (main text Figure 2B part E). Next, we randomly deleted a node from this original assortment whenever the number of nodes in the 'true' subnetwork dropped as NBS threshold stringency increased (main text Figure 2B part F). This gave us 100 thresholds for this randomly generated subnetwork, and for each we calculated the Dice Coefficient with the same combination hubs, and then calculated a single mean 'random' Dice Coefficient as before. We repeated this procedure 10,000 times yielding 10,000 random Dice Coefficients (main text Figure 2B parts G-H), which allowed us to test the significance of the true Dice Coefficient (main text Figure 2B part I). This procedure was then repeated for each of the combination hub thresholds (10-40%), thereby giving a p-value for each hub threshold.

At some more lenient network-based statistic thresholds the network-based statistic defined dopamine associated networks contained all nodes of the SAL/DMN networks. In these cases, all nodes will overlap with the hub nodes, and so it is not valid to test if overlap is statistically significant. In these cases, we increased NBS stringency until the network no longer contained all the nodes in question.

We finally examined overlap between the dopamine associated nodes identified in Experiment 1 and those identified in Experiment 2. In this case we only compared overlap at NBS thresholds where both experiments showed the same number of dopamine associated nodes. We calculated the dice coefficient between the two networks, and compare it to a null distribution generated as before.

Software

Statistical analysis was undertaken in MATLAB 2016b and R 3.3.2.

Diagrams were constructed in R using ggplot2 (2.2.1) and plotly (4.7.1).

Ball and stick networks diagrams (Figure 4A) were constructed using BrainNet viewer.[27]

Node diagram (Figure S1) was constructed using ITK-SNAP[28] and ParaView.[29]

Supplemental Results

Table S1. Correlation between rfMRI motion (mean framewise displacement) and network measures

Correlation examined	^{18}F-DOPA	^{11}C-PHNO
Motion:SAL strength	r_p=-0.17, p=0.47	r_p=0.17, p=0.43
Motion:DMN strength	r_p=0.14, p=0.52	r_p=0.03, p=0.89
Motion:(SAL-DMN) strength	r_p=-0.23, p=0.31	r_p=0.09, p=0.67
Motion: Dopamine measure	r_p=0.02, p=0.9	r_p=0.15, p=0.49

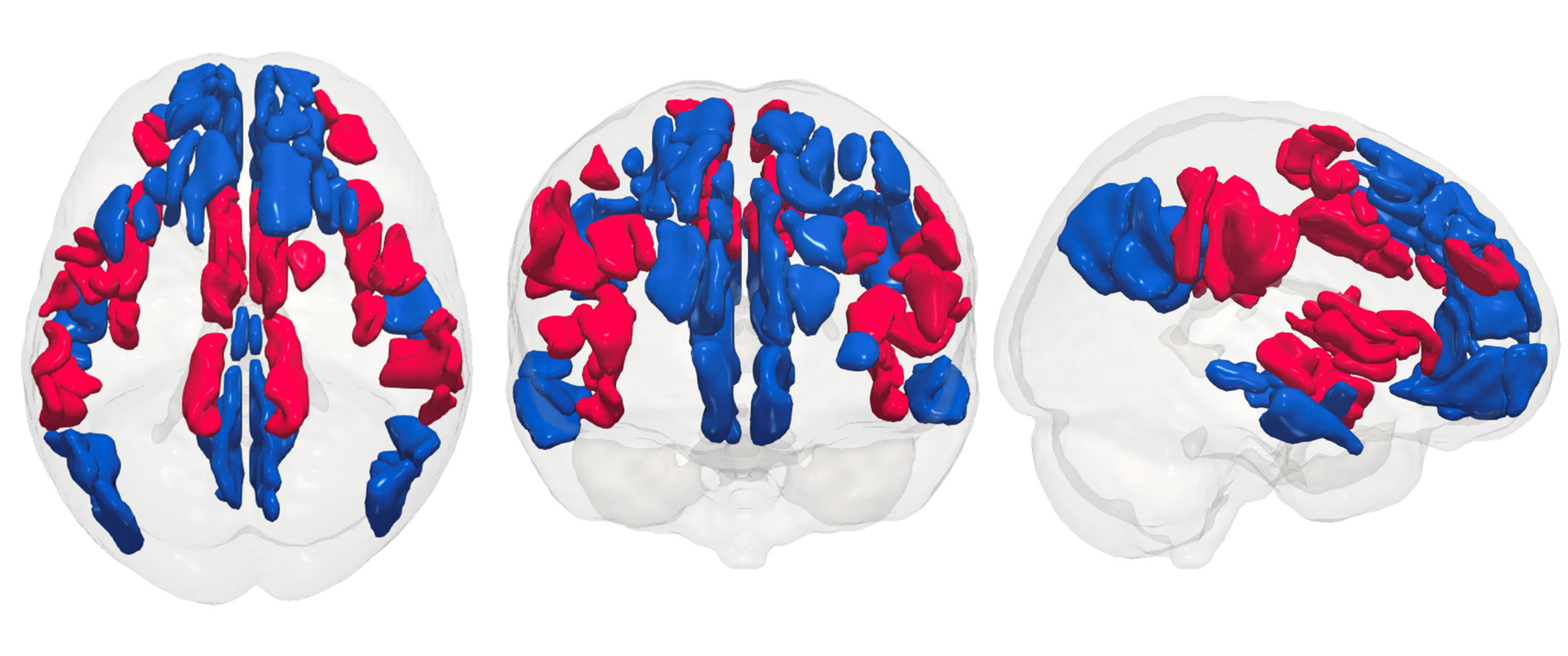

Figure S1: Salience and default mode networks of the Gordon atlas

Salience (red) and default mode (blue) network nodes of the Gordon cortical atlas

Figure S2: Correlation between average network strength (Gordon parcellation) and dopamine measures in striatal subdivisions

18F-DOPA Salience Network

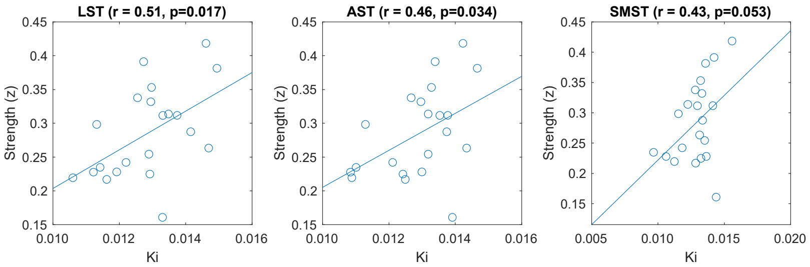

18F-DOPA Default Mode Network

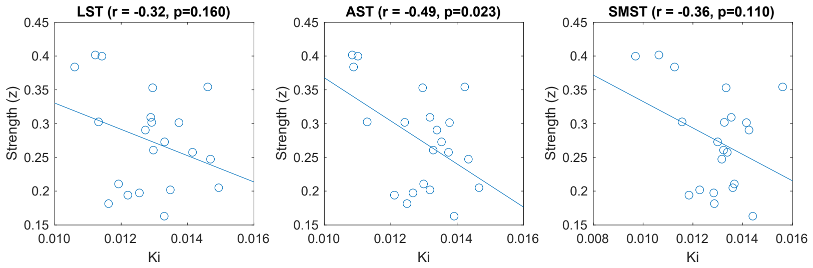

11C-PHNO Salience Network

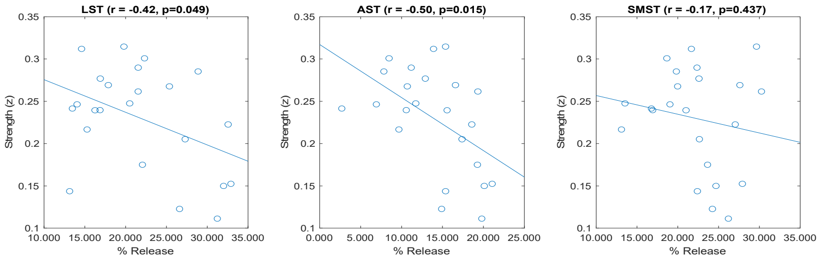

11C-PHNO Default Mode Network

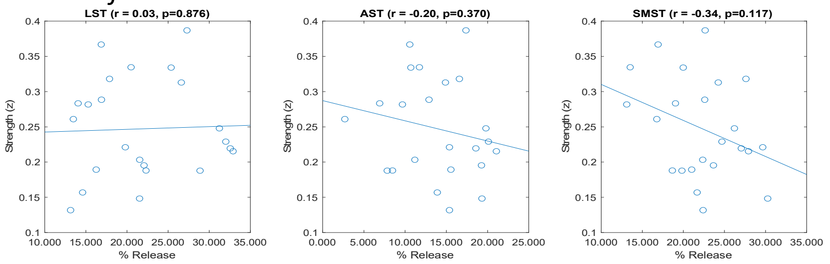

Figure S3: Correlation between limbic dopamine measures and salience network strength for a range of parcellation schemes

18F-DOPA Salience Network

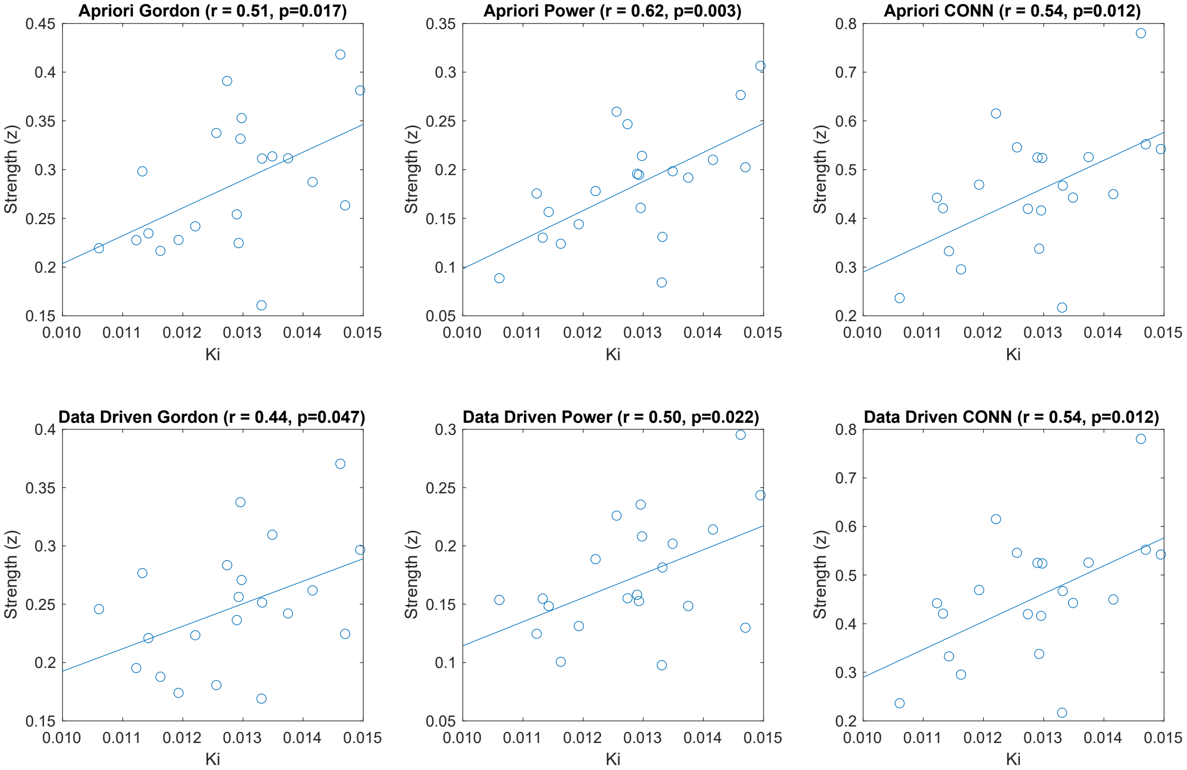

11C-PHNO Salience Network

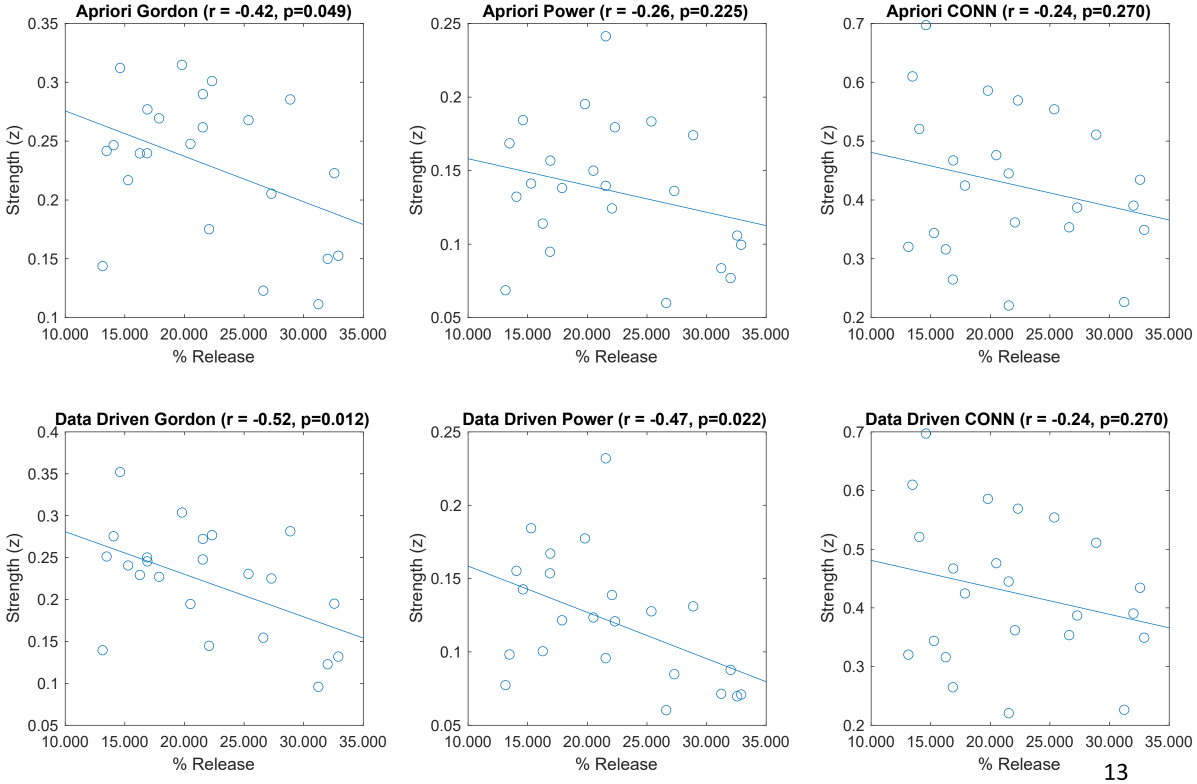

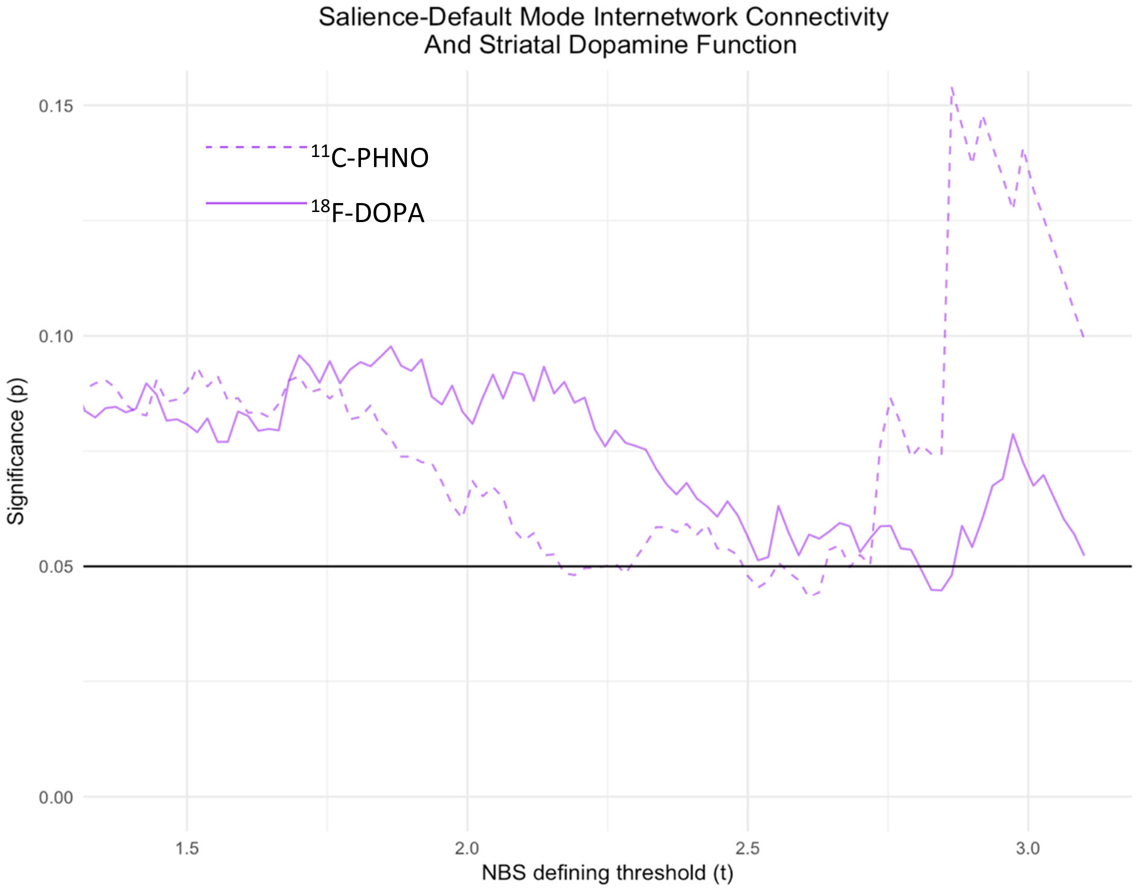

Figure S4. Relationship between salience-default internetwork connectivity and limbic dopamine function

For ^{11}C-PHNO the p-value represents the significance of the relationship between greater BPND and greater internetwork connectivity as calculated using NBS, while for ^{18}F-DOPA this refers to an association with weaker internetwork connectivity.

Figure S5: Network-based statistic results for and dopamine measures in various regions (Gordon parcellation)

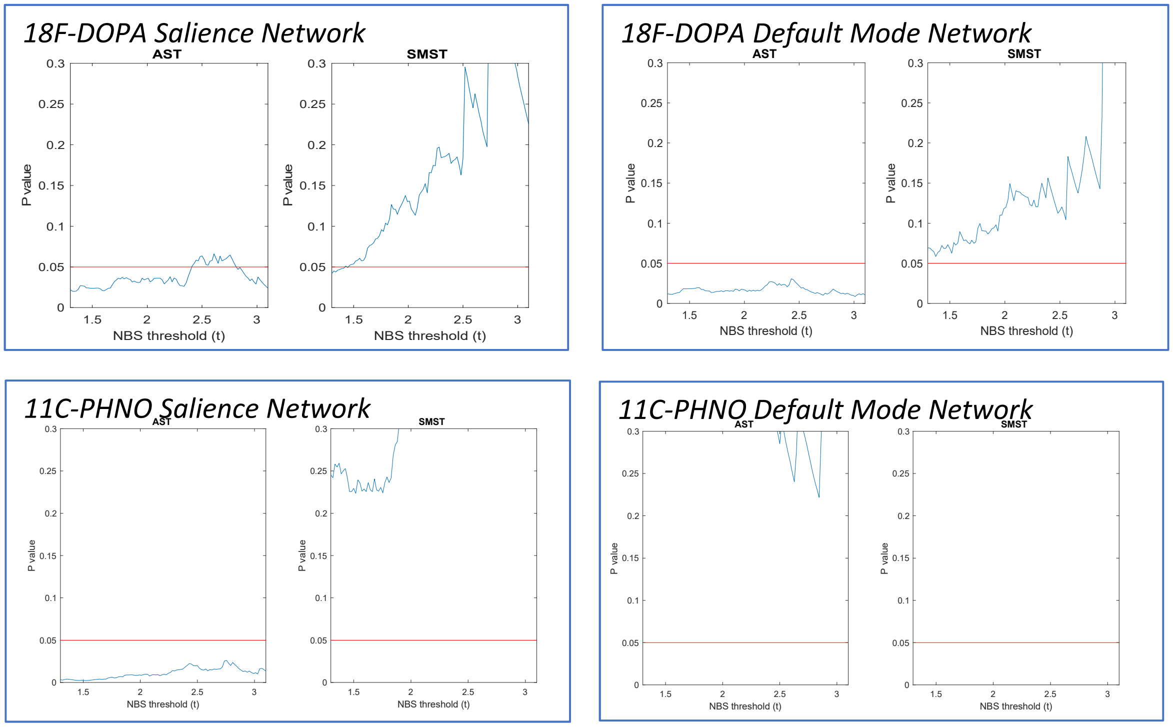

Figure S6: Network based statistic identifies subnetworks significantly associated with limbic dopamine measures for a range of parcellations

18F-DOPA Salience Network

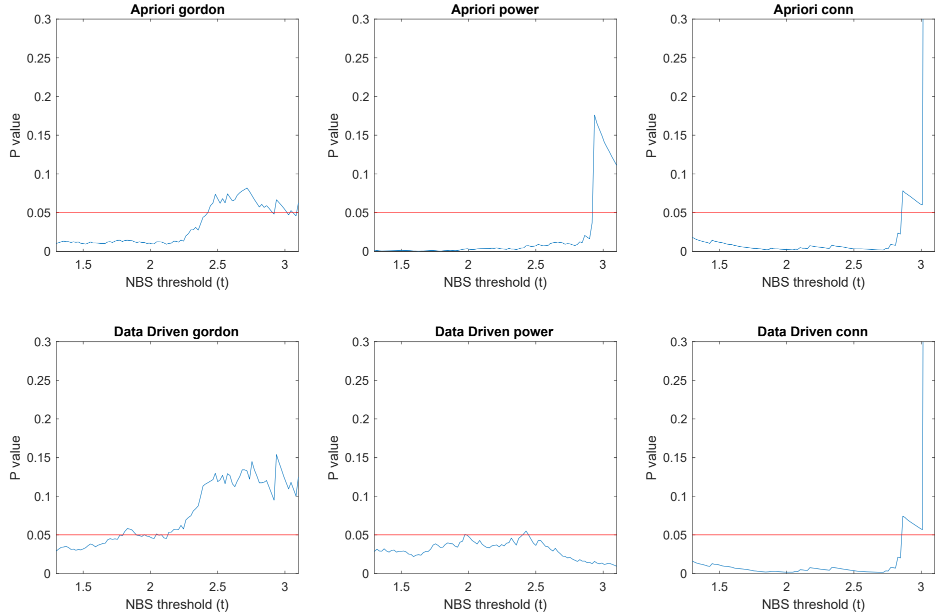

11C-PHNO Salience Network

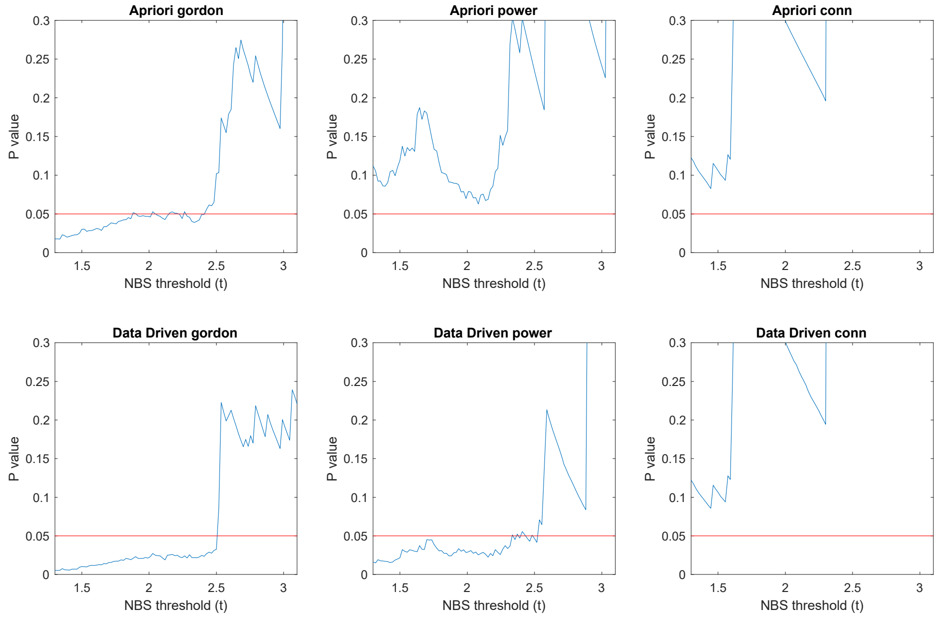

Figure S7: Significance of overlap between nodes showing strongest association with limbic dopamine measures and network combination hubs, for a range of parcellations

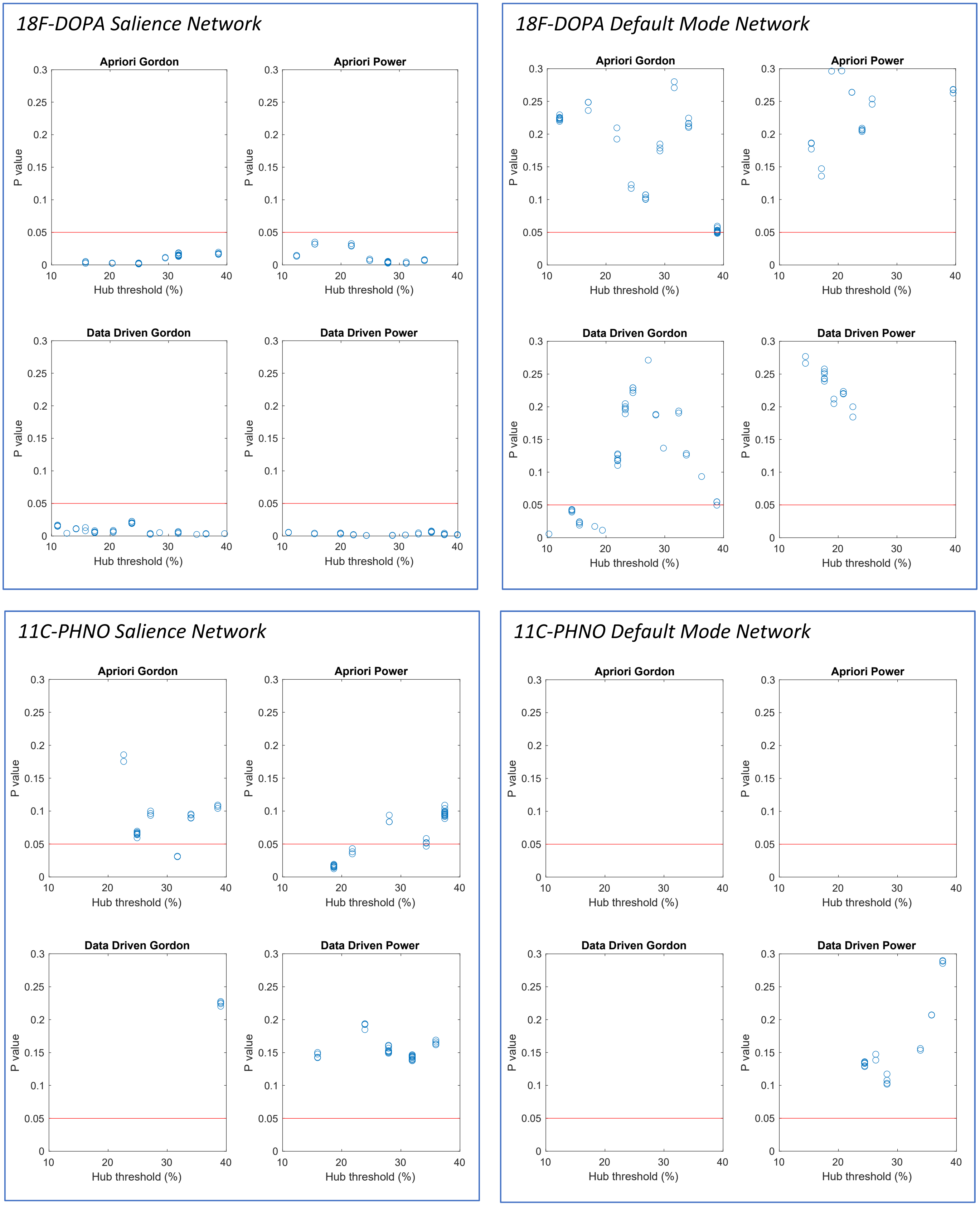

Supplemental References

1 Jauhar S, Veronese M, Rogdaki M, Bloomfield M, Natesan S, Turkheimer F *et al.* Regulation of dopaminergic function: an [18F]-DOPA PET apomorphine challenge study in humans. *Transl Psychiatry* 2017; 7: e1027.

2 Turkheimer FE, Brett M, Visvikis D, Cunningham VJ. Multiresolution analysis of emission tomography images in the wavelet domain. *J Cereb Blood Flow Metab Off J Int Soc Cereb Blood Flow Metab* 1999; 19: 1189–1208.

3 Studholme C, Hill DL, Hawkes DJ. Automated 3-D registration of MR and CT images of the head. *Med Image Anal* 1996; 1: 163–175.

4 Patlak CS, Blasberg RG. Graphical Evaluation of Blood-to-Brain Transfer Constants from Multiple-Time Uptake Data. Generalizations. *J Cereb Blood Flow Metab* 1985; 5: 584–590.

5 Gunn RN, Coello C, Searle G. Molecular Imaging And Kinetic Analysis Toolbox (MIAKAT)-A Quantitative Software Package for the Analysis of PET Neuroimaging Data. *J Nucl Med* 2016; 57: Supplement 2 1928.

6 Gunn RN, Lammertsma AA, Hume SP, Cunningham VJ. Parametric Imaging of Ligand-Receptor Binding in PET Using a Simplified Reference Region Model. *Neuroimage* 1997; 6: 279–287.

7 Lammertsma AA, Hume SP. Simplified reference tissue model for PET receptor studies. *Neuroimage* 1996; 4: 153–8.

8 Auerbach EJ, Xu J, Yacoub E, Moeller S, Uğurbil K. Multiband accelerated spin-echo echo planar imaging with reduced peak RF power using time-shifted RF pulses. *Magn Reson Med* 2013; 69: 1261–1267.

9 Cauley SF, Polimeni JR, Bhat H, Wald LL, Setsompop K. Interslice leakage artifact reduction technique for simultaneous multislice acquisitions. *Magn Reson Med* 2014; 72: 93–102.

10 Setsompop K, Gagoski BA, Polimeni JR, Witzel T, Wedeen VJ, Wald LL. Blipped-controlled aliasing in parallel imaging for simultaneous multislice echo planar imaging with reduced g-factor penalty. *Magn Reson Med* 2012; 67: 1210–1224.

11 Xu J, Moeller S, Auerbach EJ, Strupp J, Smith SM, Feinberg DA *et al.* NeuroImage Evaluation of slice accelerations using multiband echo planar imaging at 3 T. *Neuroimage* 2013; 83: 991–1001.

12 Jack CR, Bernstein MA, Fox NC, Thompson P, Alexander G, Harvey D *et al.* The Alzheimer ' s Disease Neuroimaging Initiative (ADNI): MRI Methods. *J Magn Reson Imaging* 2008; 27: 685–691.

13 Whitfield-Gabrieli S, Nieto-Castanon A. Conn: A Functional Connectivity Toolbox for Correlated and Anticorrelated Brain Networks. *Brain Connect* 2012; 2: 125–141.

14 Gordon EM, Laumann TO, Adeyemo B, Huckins JF, Kelley WM, Petersen SE. Generation and Evaluation of a Cortical Area Parcellation from Resting-State Correlations. *Cereb Cortex* 2016; 26: 288–303.

15 Power JD, Cohen AL, Nelson SSM, Wig GS, Barnes KA, Church JA *et al.* Functional network organization of the human brain. *Neuron* 2011; 72: 665–678.

16 Lord LD, Allen P, Expert P, Howes O, Lambiotte R, McGuire P *et al.* Characterization of the anterior cingulate's role in the at-risk mental state using graph theory. *Neuroimage* 2011; 56: 1531–1539.

17 Blondel VD, Guillaume JL, Lambiotte R, Lefebvre E. Fast unfolding of communities in large networks. *J Stat Mech Theory Exp* 2008; 2008: 1–12.

18 Lancichinetti A, Fortunato S. Consensus clustering in complex networks. *Sci Rep* 2012; 2. doi:10.1038/srep00336.

19 Zalesky A, Fornito A, Bullmore ET. NeuroImage Network-based statistic : Identifying differences in brain networks. *Neuroimage* 2010; 53: 1197–1207.

20 Power JD, Cohen AL, Nelson SM, Wig GS, Barnes KA, Church JA *et al.* Functional Network Organization of the Human Brain. *Neuron* 2011; 72: 665–678.

21 Rubinov M, Sporns O. NeuroImage Complex network measures of brain connectivity : Uses and interpretations. *Neuroimage* 2010; 52: 1059–1069.

22 Freeman LC. Centrality in Social Networks. *Soc Networks* 1978; 1: 215–239.

23 Power J, Schlaggar B. Evidence for hubs in human functional brain networks. *Neuron* 2013; 79: 1–29.

24 Guimera R, Nunes Amaral L. Functional cartography of complex metabolic networks. *Nature* 2005; 433: 895–900.

25 Dice LR. Measures of the Amount of Ecologic Association Between Species. *Ecology* 1945; 26: 297–302.

26 Jann K, Gee DG, Kilroy E, Schwab S, Smith RX, Cannon TD *et al.* Functional connectivity in BOLD and CBF data: SIMILARITY and reliability of resting brain networks. *Neuroimage* 2015; 106: 111–122.

27 Xia M, Wang J, He Y. BrainNet Viewer: A Network Visualization Tool for Human Brain Connectomics. *PLoS One* 2013; 8. doi:10.1371/journal.pone.0068910.

28 Yushkevich PA, Piven J, Hazlett HC, Smith RG, Ho S, Gee JC *et al.* User-guided 3D active contour segmentation of anatomical structures: Significantly improved efficiency and reliability. *Neuroimage* 2006; 31: 1116–1128.

29 Madan CR. Creating 3D visualizations of MRI data: A brief guide. *F1000Research* 2015. doi:10.12688/f1000research.6838.1.
